# In-Competition Severe Injury Events in Elite Alpine Ski Racing from 1997 to 2020: The Case of the Austrian Ski Team

**DOI:** 10.1186/s40798-021-00384-w

**Published:** 2022-01-12

**Authors:** Michael Barth, Hans-Peter Platzer, Carina Andrea Forstinger, Gunnar Innerhofer, Anton Giger, Peter Schröcksnadel, Werner Nachbauer

**Affiliations:** 1 Department of Business and Society, University of Applied Sciences Kufstein Tyrol, Kufstein, Tyrol Austria; 2 Department of Sport Science, University of Innsbruck, Innsbruck, Tyrol Austria; 3 ISAG - Institute of Sports Medicine, Alpine Medicine and Health Tourism, Hall in Tyrol, Tyrol Austria; 4 Sprachtante, Editorial Services, Innsbruck, Tyrol Austria; 5 Austrian Ski Federation, Innsbruck, Tyrol Austria

**Keywords:** Alpine ski racing, Ski racers, Injuries, World Cup, Sex differences, Disciplines

## Abstract

**Background:**

To increase safety in elite alpine ski racing Injury Surveillance Systems were implemented and preventive measures introduced. However, studies analysing the change in athletes’ injury risk by controlling for their exposure are still scarce.

**Objectives:**

This study aimed to describe and analyse the risk of in-competition severe injury events (SIE_comp_) in elite alpine ski racing.

**Methods:**

Data recorded in the Austrian Ski Federation’s Injury Surveillance System were used to analyse the SIE_comp_ incidence. Information on athletes’ competition exposure was obtained from the official website of the International Ski Federation. In 23 seasons, 2333 skier seasons were recorded for the Austrian Ski Team. Within a total of 114,531 runs 169 SIE_comp_ occurred. Generalised Estimating Equation for Poisson Regressions were applied.

**Results:**

The SIE_comp_ incidence per 1000 runs was 1.48 [95% confidence interval (CI) 1.26–1.73] for elite alpine ski racers and 2.21 (95% CI 1.79–2.75) for the subgroup of World Cup racers. A significant sex difference was detected for the subgroup of junior racers with a higher risk for female athletes [risk ratio (RR): 2.97, 95% CI 1.46–6.05]. Between the seasons of 1997 and 2020, the seasonal SIE_comp_ incidence increased by a factor of 2.67 for elite alpine ski racers and 3.53 for World Cup racers. Downhill (2.75, 95% CI 2.18–3.47) had the highest SIE_comp_ incidence, followed by super-G (1.94, 95% CI 1.30–2.88), giant slalom (1.40, 95% CI 1.06–1.85), and slalom (0.64, 95% CI 0.43–0.96).

**Conclusion:**

Although many preventive measures have been implemented in elite alpine ski racing, the risk of SIE_comp_ has increased over the last two decades.

## Key Findings and Central Implications

- Between the seasons 1997 and 2020, the seasonal incidence of severe in-competition injury events (SIE_comp_) per 1000 runs increased by a factor of 2.67 for elite alpine ski racers and 3.53 for the subgroup of World Cup racers.
- The overall incidence of SIE_comp_ per 1000 runs was 1.48 (95% CI 1.26–1.73); in downhill it was 2.75 (95% CI 2.18–3.47), in super-G 1.94 (95% CI 1.30–2.88), in giant slalom 1.40 (95% CI 1.06–1.85), and in slalom 0.64 (95% CI 0.43–0.96).
- Despite the introduction of many preventive measures—particularly in FIS Alpine Ski World Cup competitions—our results revealed a still rising risk of SIE_comp_, calling for further efforts by all stakeholders (i.e. ski federations, equipment suppliers, researchers) to search for causes for the ongoing incidence increase and develop effective measures for injury prevention.

## Introduction

### Problem Statement and Research Questions

Over the past 20 years, many preventive measures such as competition equipment regulations or safety gate panels have been introduced to reduce the injury risk in alpine ski racing. To monitor ski racing-related injury trends, compare the discipline-specific injury incidence, and determine potential risk factors, the Austrian Ski Federation as well as the International Ski Federation (FIS) implemented Injury Surveillance Systems (ISSs). In this context, one of the major challenges is the definition of injuries, especially injury severity, which has varied across available ISSs data and reported results. As the extent to which athletes participate in training and competitions affects their risk of injury, defining and collecting meaningful exposure data is as important as collecting information about the injuries. In general, exposure is quantified as the time during which athletes are at risk [1]. In ski racing, skier seasons and the number of runs in competitions and training have been used to assess the exposure.

When calculating injury incidence, one should consider the numerator and denominator in detail. For this contribution—and thus in the context of elite alpine ski racing—we formulated the three following research questions:

- *Q1* What are the injury incidence (per 100 skier seasons and per 1000 runs) and the respective sex-specific risk ratio (RR) in elite alpine ski racing?
- *Q2* How did the injury incidence (per 100 skier seasons and per 1000 runs) in elite alpine ski racing change over time?
- *Q3* What are the injury incidence (per 1000 runs) and the respective sex-specific RR in elite alpine ski racing’s four major disciplines—i.e. downhill, super-G, giant slalom, and slalom?

In answering each of these questions, we distinguished between all injuries and severe injuries only.

### State of Research and Objective of the Empirical Investigation

In a comprehensive literature review, Barth et al. [2] analysed original epidemiological studies on injuries in FIS Alpine Ski World Cup (WC) and FIS Alpine Skiing Europa Cup (EC) racing by applying the consensus statement for the ‘recording and reporting of epidemiological data on injury and illness in sport 2020’ [1] as an analysis grid. We based our literature review on Barth et al. [2] but expanded their work with new analyses. Table 1 describes central characteristics of currently available studies.

**Table 1:** Epidemiological studies on injuries in WC and EC racing (adapted and extended from Barth et al. [2])

	Flørenes et al. [3]	Bere et al. [4]	Haaland et al. [5]	Alhammoud et al. [6]	Barth et al. [2]
Design	Retrospective cross-sectional	Retrospective cross-sectional	Retrospective cross-sectional	Prospective cohort study	Prospective cohort study
Data from FIS ISS	Yes	Yes	Yes	No	No
Country/countries	Austria, Canada, Finland, France, Germany, Italy, Norway, Sweden, Switzerland, Slovenia (2008 only)	Ten nations (see ref [3])	International, N/A	France	Austria
Sample	WC racers	WC racers	WC racers	EC racers	Elite alpine ski racers in the Austrian ski team
Covering of season (according to authors’ descriptions)	WC competition season (starting on 1 November or, if earlier, the 1st WC competition of the season, until the 2 final WC competitions)	WC competition season (during the 5-month WC season)	WC competition season (1st to last WC events)	Complete season (1 May–30 April)	Complete season (1 May–30 April)
Period (from season^a^ to season^a^)	2007–2008	2007–2012	2007–2015	2014–2018	1997–2018, without 1999
Seasons (n)	2	6	9	5	22
Type of sport activity	Competition, on-snow training, off-snow training	Competition, on-snow training^b^	Competition, on-snow training	Competition, on-snow training, off-snow training	Competition, on-snow training
Mode of injury onset (presentation)	Sudden onset	Sudden onset	Sudden onset	Sudden onset, gradual onset	Sudden onset
Mode of injury onset (mechanism)	Acute	Acute	Acute	Acute, repetitive	Acute
Definition of severe injury	> 28 days of absence from training and competition	> 28 days of absence from training and competition	> 28 days of absence from training and competition	> 28 days of absence from training and competition	> 35 days of absence from training and competition
Multiple injury count method	One body part per event	N/A	N/A	One body part per event	Distinctive analysis

EC, FIS Alpine Skiing Europa Cup; FIS ISS, International Ski Federation Injury Surveillance System; N/A, no data available; WC, FIS Alpine Ski World Cup

^a^Season expressed with the ending year (e.g. season 2006/07 expressed as 2007)

^b^Classification due to the corresponding numbers of Haaland et al. [5], who restricted training to ‘official training, team training and other activity on snow, for example, free skiing or warm up’

Previous studies have already examined the injury incidence and respective sex-specific RR in elite alpine ski racing, which are the subject of *Q1*. We summarised the findings in Table 2.

**Table 2:** Incidence of (severe) injuries per 100 skier seasons and per 1000 runs and sex-specific RR

Injury severity category	Sex	Measure	Flørenes et al. [3]	Bere et al. [4]	Haaland et al. [5]	Alhammoud et al. [6]	Barth et al. [2]
Incidence per 100 skier seasons							
All severities	Total	Incidence	36.7	36.2	33.1	73.7 (124.8)	N/A
	Male	Incidence	42.1	39.7	35.7^b^	70.9 (121.5)	N/A
	Female	Incidence	29.7	31.9	29.7^b^	77.8 (129.6)	N/A
	Male/female	RR	1.42*	1.24*	1.20*^b^	0.91^b^ (0.94^b^)	N/A
	Female/male	RR	0.70*^b^	0.80*^b^	0.83*^b^	1.10^b^ (1.07^b^)	N/A
Severe injuries	Total	Incidence	11.3	12.9	12.9	26.3 (38.3)	11.5 (15.7)^a^
	Male	Incidence	11.6	13.7	N/A	N/A	9.7 (12.2)^a^
	Female	Incidence	10.9	12.0	N/A	N/A	13.6 (20.1)^a^
	Male/female	RR	1.07	1.14	N/A	N/A	0.72*^a^ (0.61*^a^)
	Female/male	RR	0.94^b^	0.88^b^	N/A	N/A	1.39*^a^ (1.65*^a^)

Incidence per 1000 runs							
Level of competition			WC/WSC competitions	WC/WSC/WOG competitions	WC/WSC/WOG competitions	N/A	N/A
All severities	Total	Incidence	9.8	9.3	8.7^b^	N/A	N/A
	Male	Incidence	12.7	11.3	10.2^b^	N/A	N/A
	Female	Incidence	6.2	7.1	7.0^b^	N/A	N/A
	Male/female	RR	2.05*	1.58*	1.46*^b^	N/A	N/A
	Female/male	RR	0.49*^b^	0.63*^b^	0.68*^b^	N/A	N/A
Severe injuries	Total	Incidence	N/A	2.9	3.0^b^	N/A	N/A
	Male	Incidence	N/A	3.6	N/A	N/A	N/A
	Female	Incidence	N/A	2.1	N/A	N/A	N/A
	Male/female	RR	N/A	1.70*	N/A	N/A	N/A
	Female/male	RR	N/A	0.59*^b^	N/A	N/A	N/A

N/A, no data available; RR, risk ratio; WC, FIS Alpine Ski World Cup; WOG, Winter Olympic Games; WSC, FIS Alpine World Ski Championships

For Alhammoud et al. [6] and Barth et al. [2]: figures without parenthesis: incidence of in-competition season (severe) injuries; figures in parenthesis: incidence of in- and out-of-competition season (severe) injuries

* Sig. result in the risk ratio of male vs. female athletes

^a^Information provided by the authors

^b^Calculated based on article information

As shown in Table 2, studies referring to elite alpine ski racing—i.e. studies including WC racers in their samples—reported an injury incidence per 100 skier seasons between 33.1 and 36.7; for severe injuries, this incidence was between 11.3 and 12.9. The injury incidence per 1000 runs was between 8.7 and 9.8; however, with Bere et al. [4] and Haaland et al. [5] only two studies specifically analysed severe injuries, reporting an incidence of 2.9 and 3.0. Interestingly, the injury incidence per 1000 runs analyses were all restricted to WC, FIS Alpine World Ski Championships, and Winter Olympic Games competitions. However, some WC athletes also race in lower-level competitions like the EC within one competition season. Hence, we have no information on the in-competition (severe) injury incidence per 1000 runs in male and female elite alpine ski racers covering all FIS competitions elite alpine ski racers participate in during a whole season.

Concerning *Q2*, which is focused on the change in the injury incidence over time, literature is scarce. In fact, only Barth et al. [2] included WC racers and applied regression analysis to study the injury incidence change over the course of time. They calculated the average seasonal growth rate for the on-snow severe injury event incidence (per 100 skier seasons) and reported values of 0.49 for WC racers and 0.57 for EC racers, respectively; however, it has to be said that they did not correct for the extent of athletes’ participation—i.e. the number of runs. Although Bere et al. [4] as well as Haaland et al. [5] graphically showed the change in the seasonal (time-loss) injury incidence per 1000 runs, they did not apply any approach to further quantify a possible decrease or increase in the seasonal injury incidence. Hence, no information on the change in the seasonal severe injury incidence per 1000 runs in elite alpine ski racing is available at the moment.

*Q3* focuses on the injury incidence in elite alpine ski racing’s four major disciplines. Findings from previous studies are depicted in Table 3.

**Table 3:** Injury incidence per 1000 runs and respective sex-specific RR in downhill, super-G, giant slalom, and slalom

Discipline	Study	Total	Male	Female	RR
Downhill	Flørenes et al. [3]	17.2	19.3	13.9	1.39
	Bere et al. [4]	18.1	20.6	14.5	1.42
	Haaland et al. [5]	16.6^a^	N/A	N/A	N/A
Super-G	Flørenes et al. [3]	11.0	14.5	7.7	1.90
	Bere et al. [4]	11.1	14.0	8.2	1.70
	Haaland et al. [5]	10.9^a^	N/A	N/A	N/A
Giant slalom	Flørenes et al. [3]	9.2	12.8	5.1	2.51
	Bere et al. [4]	8.0	10.8	5.2	2.07*
	Haaland et al. [5]	7.8^a^	N/A	N/A	N/A
Slalom	Flørenes et al. [3]	4.9	7.5	1.5	5.16*
	Bere et al. [4]	4.0	4.0	4.1	0.98
	Haaland et al. [5]	3.4^a^	N/A	N/A	N/A

RR, risk ratio

* Sig. result in the RR of male versus female athletes

^a^Calculated based on article information

In the context of *Q3*, a clear research gap emerged: At the moment, there is no information on the discipline-specific incidence of severe injuries per 1000 runs and accordingly on the respective sex-specific RR.

To address the identified research gaps, the objective of this study’s empirical investigation was threefold: first, describing the incidence per 1000 runs and sex-specific RR of in-competition severe injury events (SIE_comp_) in elite alpine ski racing; second, examining the change in the seasonal SIE_comp_ incidence per 1000 runs over 24 seasons; and third, comparing the SIE_comp_ incidence per 1000 runs and the respective sex-specific RR in downhill, super-G, giant slalom, and slalom.

## Methods

### Data Collection

Injury data used for the analysis stem from the Austrian Ski Federation’s ISS, which was already established in 1993. Until 2009, data collection was done by using a paper-and-pencil questionnaire. Since then, an adapted online version of this instrument has been applied and continuously developed further (e.g. integration of an atlas of anatomy). To reduce a potential recall bias, all team members were advised to record injuries immediately after occurrence. At the end of each season records were checked (for more details see Barth et al. [2]). Due to methodological issues, data from the seasons 1993 to 1996 as well as 1999 had to be discarded; thus, data from 23 seasons over a time span of 24 seasons were analysed. A season lasted from 1 May to 30 April of the following year.

Between the seasons 1997 and 2020, 2333 skier seasons (1291 of male athletes; 1042 of female athletes) were recorded for the Austrian Ski Team and can be assigned to its four different squads: Team National (*n* = 501), Team A (*n* = 462), Team B (*n* = 732), and Team C (*n* = 638).

The criteria used to ascribe athletes to Team National, Team A, and Team B were primarily based on the World Cup Start List (WCSL), i.e. the points systems used to determine the start positions at races. Team National consists of the best racers (e.g. WCSL top 10, medal winner at Olympic Games or World Championships). A WCSL position from 11 to 15 qualifies a racer for Team A. For Team B not only the WCSL position, but also age is considered as qualification criterion. The qualification criteria for Team C are also based on competition results. Although further criteria (e.g. EC ranking, FIS ranking, coach judgment) are used and criteria were slightly adjusted between 1997 and 2020, it seems justified to conclude that Team National, Team A, and Team B racers represent some of the best ski racers in the World and in Europe; Team C comprises some of the best junior racers.

Injury events were classified as severe when the absence from training and/or competition lasted longer than five weeks. Since data on the number of training runs were not available for all seasons, we had to restrict our analysis to severe injury events occurring during FIS competitions, including the official downhill training runs (hereinafter: SIE_comp_). Injury data from all seasons from 1997 to 2020 except 1999 were available for the analysis. Of the 371 severe injury events that occurred, 169 were classified as SIE_comp_.

Information on every athlete’s exposure to risk in terms of participation in FIS competitions including the official downhill training runs was obtained from the official FIS website [7]. We counted the number of runs (and not the number of competitions), which means that giant slalom as well as slalom competitions counted as two runs if an athlete managed to qualify for and started in the second run. When an athlete did not finish a run or was disqualified, we counted the respective run as a full run. In combined competitions, we assigned the runs as well as injuries that occurred to the respective disciplines. Until the season 2007/08 (hereinafter: 2008), official downhill training runs were not included in the FIS database. Thus, we calculated sex- and team-specific ratios of competitions and official trainings from the season 2008 onwards to obtain multiplicators for the seasons before 2008. The respective multipliers were then used to calculate an athlete’s downhill exposure—i.e. the number of runs in competitions and official training an athlete did.

In sum, Austrian Ski Team athletes participated in 114,531 runs (male athletes: 64,395; female athletes: 50,137; Team National: 18,153; Team A: 17,978; Team B: 39,226; Team C: 39,174). For further analyses, we pooled Team National and Team A as Team World Cup (Team WC). Team B—consisting largely of racers competing in the EC—is hereinafter referred to as Team Europa Cup (Team EC). Team C comprised junior alpine ski racers and is hereinafter called Team National Junior (Team NJ).

### Data Analysis

Following the approach of the previous empirical studies (see Tables 1, 2, 3), we expressed injury incidence as the number of SIE_comp_ per 100 skier seasons or per 1000 runs. We applied Generalised Estimating Equation for Poisson Regression (package gee, version 4.13–20 [8]), which allowed us to take into account the dependence within clusters—i.e. the fact that athletes are commonly members of the Austrian Ski Team for several years and therefore the measurements for one and the same athlete were dependent. For an easier interpretation, estimated coefficients and their respective 95% (Wald) confidence intervals (CIs) were exponentiated.

To describe the change in the SIE_comp_ incidence throughout the time span of 24 seasons (using data from 23 seasons) as well as to assess the models’ adequacy [9] we made two graphical representations of the predictions, their 95% CIs (package stats, version 4.0.2 [10]), and the observations concerning the SIE_comp_ incidence (expressed as SIE_comp_ per 100 skier seasons and 1000 runs) in each season for all Austrian Ski Team racers as well as for the subgroup Team WC.

The value of the exponentiated coefficients corresponded to the sex-specific RR, which was reported as female/male ratio. Incidence and RR were presented with their 95% CIs. A two-tailed *p* ≤ 0.05 was considered significant. Implementation of all analysis was done with R (version 4.0.2).

## Results

### SIE_comp_ Incidence and Corresponding Sex-Specific RR

During 23 seasons, 169 SIE_comp_ were recorded. Table 4 displays the SIE_comp_ incidence per 1000 runs and the corresponding sex-specific RR in total as well as for Team WC, Team EC, and Team NJ.

**Table 4:** Team-specific results: number of runs, number of SIE_comp_, SIE_comp_ incidence per 1000 runs, and RR with 95% CI

	Total			Male			Female			
	Runs (*n*)	SIE_comp_ (*n*)	Incidence (95% CI)	Runs (*n*)	SIE_comp_ (*n*)	Incidence (95% CI)	Runs (*n*)	SIE_comp_ (*n*)	Incidence (95% CI)	Female–male RR (95% CI)
Total	114,531	169	1.48 (1.26–1.73)	64,395	82	1.27 (1.01–1.60)	50,137	87	1.74 (1.39–2.16)	1.36 (0.99–1.87)
Team WC	36,131	80	2.21 (1.79–2.75)	19,692	42	2.13 (1.57–2.89)	16,440	38	2.31 (1.71–3.13)	1.08 (0.71–1.67)
Team EC	39,226	51	1.30 (0.97–1.73)	22,049	28	1.27 (0.86–1.86)	17,178	23	1.34 (0.87–2.07)	1.05 (0.59–1.88)
Team NJ	39,174	38	0.97 (0.70–1.35)	22,655	12	0.53 (0.29–0.96)	16,519	26	1.57 (1.06–2.33)	2.97 (1.46–6.05)

CI, confidence interval; RR, risk ratio; SIE_comp_, in-competition severe injury events; Team EC, Team Europa Cup; Team NJ, Team National Junior; Team WC, Team World Cup

In total, the SIE_comp_ incidence per 1000 runs was 1.48 (95% CI 1.26–1.73). The RR was 1.36 with a 95% CI of 0.99–1.87, which means the difference between female and male racers was close to falling below the statistical significance threshold of 5% (*p* = 0.06). When additionally controlling for age in the regression, the empirical *p* value was below 5%; a significant sex difference, with female ski racers showing a higher injury risk than their male counterparts (1.46, 95% CI 1.07–2.00, *p* = 0.02).

Analysing the respective teams yielded more differentiated results: Neither for Team WC nor for Team EC a significant sex difference was detected (Team WC: *p* = 0.71; Team EC: *p* = 0.86; see Table 4; controlling for age, the respective *p* values were 0.72 and 0.63). In Team NJ the SIE_comp_ incidence was significantly (*p* < 0.01) higher in female ski racers compared to their male counterparts. Controlling for athletes’ age did hardly change this result (RR = 2.94, 95% CI 1.46–5.89, *p* < 0.01).

### Change in the Seasonal SIE_comp_ Incidence

**Fig. 1:**
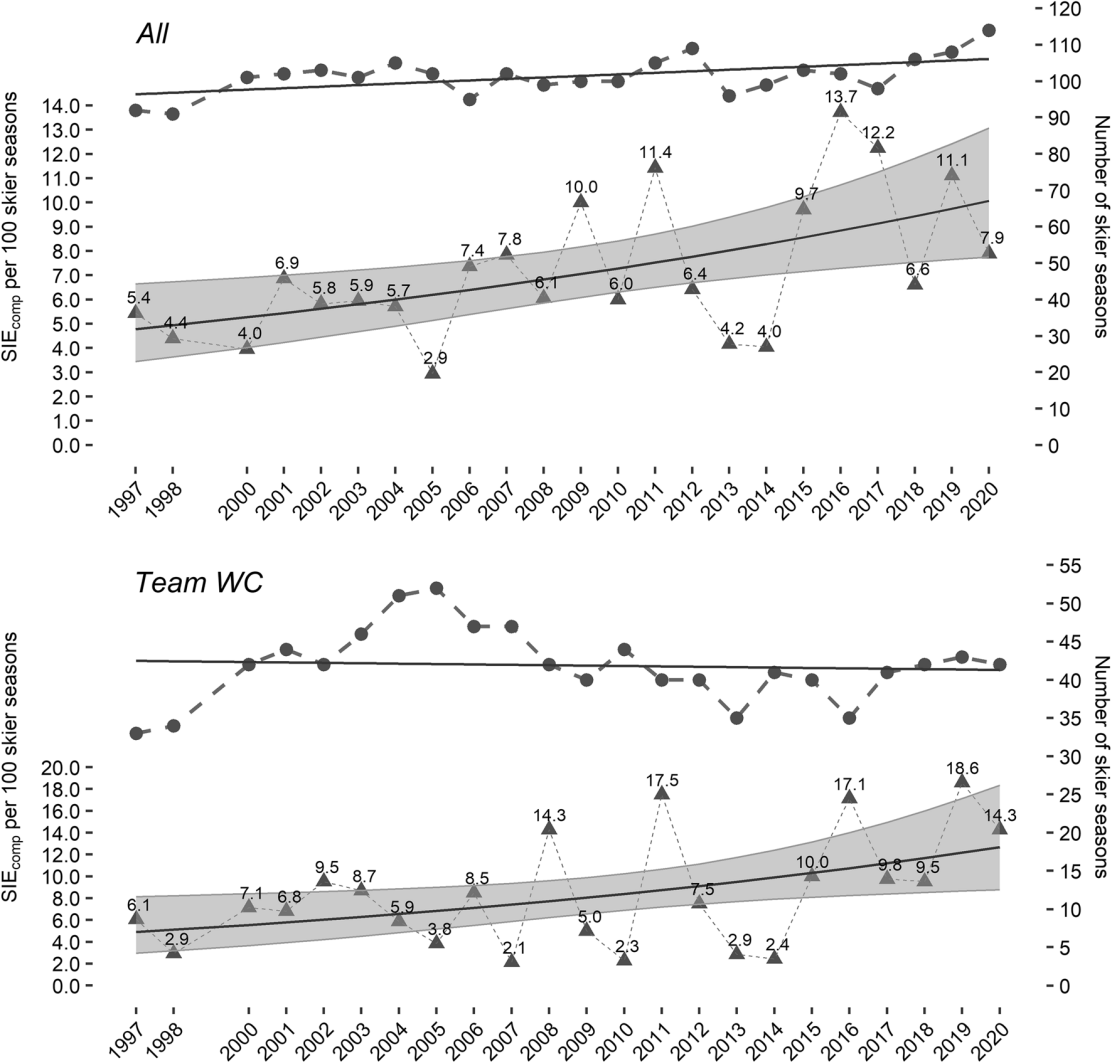
Number of skier seasons (●), seasonal SIE_comp_ incidence per 100 skier seasons (▲), and predictions (▬) with 95% CI for all Austrian Ski Team racers and Team WC. Note. SIE_comp_, in-competition severe injury events; WC, FIS Alpine Ski World Cup

Figure 1 displays the number of skier seasons and the seasonal SIE_comp_ incidence per 100 skier seasons for all racers in the Austrian Ski Team as well as for the subgroup Team WC from the seasons 1997 to 2020.

The average seasonal growth of the SIE_comp_ incidence per 100 skier seasons (predictions for seasons 1997–2020) was computed to be 0.22 for all Austrian Ski Team racers and 0.32 for Team WC. This means that the seasonal SIE_comp_ incidence for all ski racers in the Austrian Ski Team doubled (2.11) between the seasons of 1997 and 2020. The respective factor for Team WC was 2.59.

**Fig. 2:**
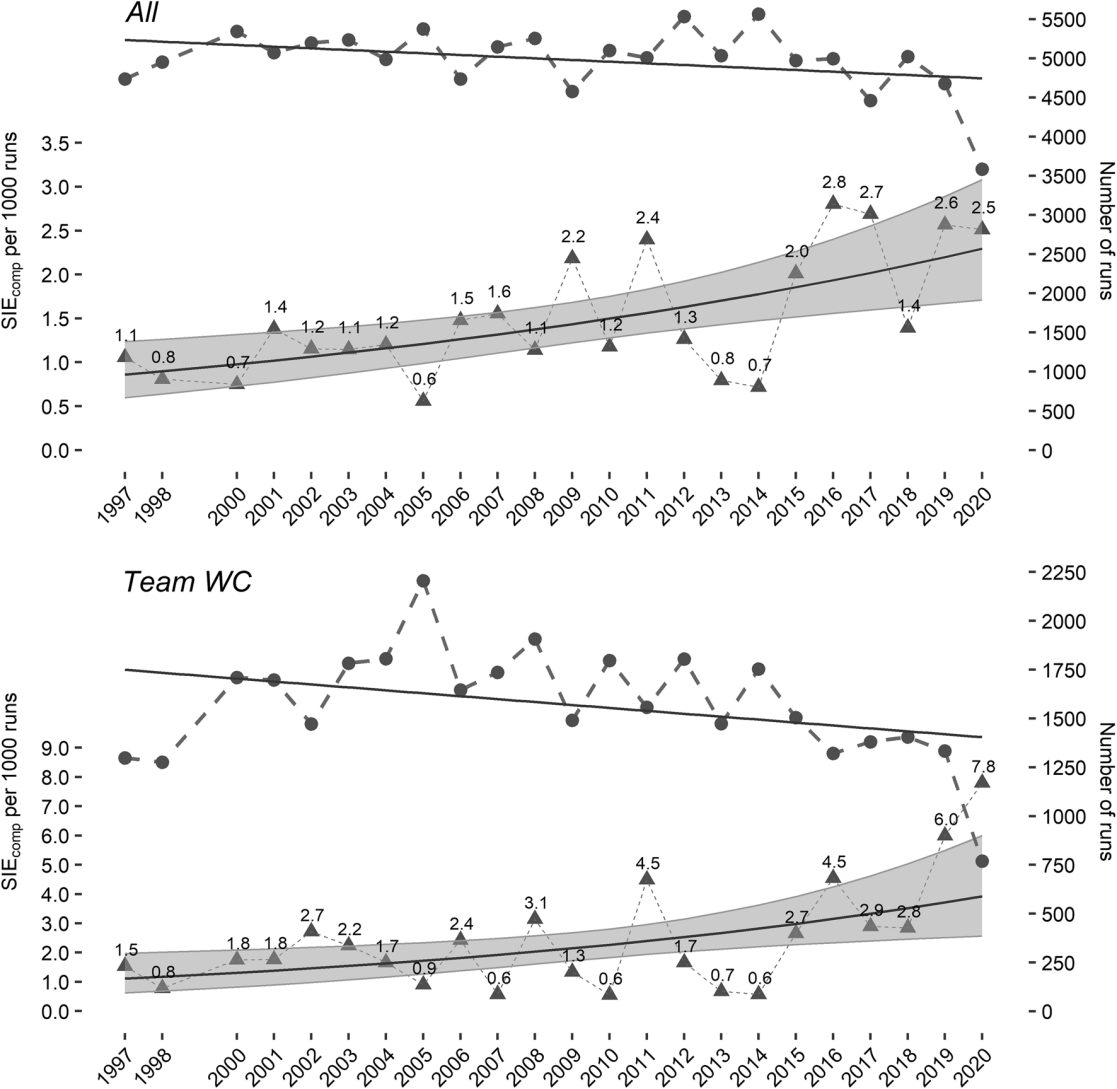
Number of runs per season (●), seasonal SIE_comp_ incidence per 1000 runs (▲), and predictions (▬) with 95% CI for all Austrian Ski Team racers and Team WC

Figure 2 displays the number of runs per season and the seasonal SIE_comp_ incidence per 1000 runs for all racers in the Austrian Ski Team as well as for the subgroup Team WC.

The average seasonal growth of the SIE_comp_ incidence per 1000 runs (using predictions for seasons 1997 and 2020) was 0.06 for all Austrian Ski Team racers and 0.12 for Team WC. This means that the seasonal SIE_comp_ incidence increased by a factor of 2.67 for all Austrian Ski Team racers and by a factor of 3.53 for Team WC between the seasons 1997 and 2020.

### Discipline-Specific SIE_comp_ Incidence Per 1000 Runs and Sex-Specific RR

While no significant (*p* = 0.44) difference in the SIE_comp_ incidence was detected between downhill (2.75, 95% CI 2.18–3.47) and super-G (1.94, 95% CI 1.30–2.88), the incidence for giant slalom (1.40, 95% CI 1.06–1.85) was significantly (*p* < 0.01) lower than that for downhill. Moreover, the SIE_comp_ incidence per 1000 runs was significantly lower in slalom (0.64, 95% CI 0.43–0.96) compared to all other disciplines (downhill: *p* < 0.01; super-G: *p* < 0.01; giant slalom: *p* = 0.01). Descriptively, all RR were above 1.0; however, we could not detect any significant sex-specific difference (downhill: *p* = 0.24; super-G: *p* = 0.24; giant slalom: *p* = 0.21; slalom: *p* = 0.80). In this context, the lower number of injuries in the sex- and discipline-specific subsamples (see Table 5) should be considered. Table 5 displays the total number of runs and SIE_comp_ as well as the SIE_comp_ incidence and the respective RR in male and female Austrian Ski Team racers, separated into the four major disciplines.

**Table 5:** Discipline-specific results: number of runs, number of SIE_comp_, SIE_comp_ incidence per 1000 runs, and RR with 95% CI

	Total			Male			Female			
	Runs (*n*)	SIE_comp_ (*n*)	Incidence (95% CI)	Runs (*n*)	SIE_comp_ (*n*)	Incidence (95% CI)	Runs (*n*)	SIE_comp_ (*n*)	Incidence (95% CI)	Female–male RR (95% CI)
Downhill	22,200	61	2.75 (2.18–3.47)	12,824	31	2.42 (1.75–3.35)	9377	30	3.20 (2.30–4.46)	1.32 (0.83–2.11)
Super-G	13,419	26	1.94 (1.30–2.88)	7300	11	1.51 (0.82–2.77)	6119	15	2.45 (1.45–4.14)	1.63 (0.73–3.63)
Giant slalom	41,482	58	1.40 (1.06–1.85)	23,036	27	1.17 (0.76–1.81)	18,446	31	1.68 (1.17–2.41)	1.43 (0.82–2.52)
Slalom	37,430	24	0.64 (0.43–0.96)	21,235	13	0.61 (0.34–1.11)	16,195	11	0.68 (0.39–1.17)	1.11 (0.50–2.48)

CI, confidence interval; RR, risk ratio; SIE_comp_, in-competition severe injury events

## Discussion

In this study we first described the SIE_comp_ incidence per 1000 runs for elite alpine ski racers and calculated the sex-specific RR. Second, we analysed the change in the seasonal SIE_comp_ incidence in alpine ski racing over more than two decades. Third, the SIE_comp_ incidence in elite alpine ski racing’s four major disciplines—downhill, super-G, giant slalom, and slalom—was determined and compared.

Referring to *Q1*: The only comparable information in the context of SIE_comp_ incidence per 1000 runs stems from the studies of Bere et al. [4] and Haaland et al. [5], which both refer to WC racers (see Tables 1, 2). With an SIE_comp_ incidence of 2.21 (95% CI 1.79–2.75) for WC racers, our results do not significantly differ from those of Bere et al. [4] (2.9, 95% CI 2.2–3.5) and Haaland et al. [5] (3.0, 95% 2.5–3.5; calculated by authors).

When additionally controlling for age in the regression analysis, we found a significant sex-related difference in elite alpine ski racers. However, this result must be viewed and interpreted in a differentiated manner. We did not observe a sex difference in the subgroup of WC racers (RR 1.08, 95% CI 0.71–1.67); a result contradicting the up to now only available result, which is that of Bere et al. [4], who identified a higher risk for male than for female WC racers (RR male/female: 1.70, 95% CI 1.07–2.70). Also for EC racers no sex-specific difference was found. However, we detected a significant sex-specific difference for the subgroup of elite junior alpine ski racers with female athletes showing an alarming three times higher injury risk (RR 2.97, 95% CI 1.46–6.05) than their male counterparts. All subgroup results were constant when additionally controlling for athletes’ age in our regression analyses.

Referring to *Q2*: From season 1997 to 2020, the SIE_comp_ incidence per 1000 runs increased for elite alpine ski racers by the factor 2.67 and for WC racers by the factor 3.53; the SIE_comp_ incidence per 100 skier seasons increased by the factor 2.11 for elite alpine ski racers and by the factor 2.59 for WC racers. Thus, for both analysed groups, the incidence per 1000 runs is higher compared to the incidence per 100 skier seasons. This shows that the increase in SIE_comp_ is not caused by athletes’ increased competition participation.

Referring to the *Q3*: Downhill showed the highest SIE_comp_ incidence per 1000 runs, followed by super-G, giant slalom, and slalom; the incidence was significantly lower in slalom than in all other disciplines and in giant slalom compared to downhill and super-G. The lower risk in slalom is partly in line with previous studies that analysed all injury severities [4, 5]. The fact that we did not detect significant sex-specific differences in any of the four disciplines should be assessed against the background of the subsamples’ low numbers of injuries. On a descriptive level, it was again remarkable that the sex-specific RR pointed to a higher risk for female than for male athletes in all disciplines, which is contrary to other studies [3, 4].

One of this study’s major strengths is that we were able to collect and incorporate appropriate individual-level data for athletes’ exposure—i.e. the amount of competition participation per athlete per season. Furthermore, these worldwide unique data on injuries in elite alpine ski racing comprise standardised and thus comparable information collected over more than two decades.

However, this study is not without limitations. We applied a prospective cohort study design with data stemming from only one national ski federation. This may limit the generalisability of the study results. Moreover, we want to stress that especially the results of our subgroup analyses should be treated with caution due to the small samples in terms of severe injuries. Unfortunately, training data for a determination of athletes’ exposure to injuries were not available for the whole period of time. Thus, we could analyse only 46% of all recorded severe injury events, which reduces our possibilities for (further) analysis. Furthermore, we defined severe injuries with an absence period of five weeks (instead of the commonly used period of four weeks); thereby, we follow Barth et al. [2] and stick to the definition the Austrian Ski Federation has continuously been using since the introduction of its ISS in 1993. However, the higher strictness of our definition of severe injuries limits the comparability of our results only slightly: There were only very few cases of injuries that caused an absence of four but not 5 weeks. Finally, we want to stress at this point that our models were not set up to predict the seasonal incidence, but to assess the central trend. We would like to encourage researchers to conduct studies on other nations’ ski teams to gain more insights into this important field of research. Collecting meaningful exposure data in competitions and training is as important as collecting information about the injuries.

## Conclusion

The main conclusion of this study is that in spite of the many efforts to reduce injury risk in alpine ski racing, SIE_comp_ risk has increased over the last 20 years. In alpine ski racing—and particularly in FIS Alpine Ski World Cup competitions—many preventive measures have been introduced in the last years. Nevertheless, our results revealed a still rising risk of SIE_comp_. This calls for further efforts by all stakeholders (i.e. ski federations, equipment suppliers, researchers) to search for causes for the ongoing incidence increase and develop effective measures for injury prevention, including the evaluation of existing measures.

## Data Availability

All data relevant to the study are included in the article or uploaded as supplementary information. All data generated or analysed during this study are included in this published article.
